# Estimating Vitamin C Intake Requirements in Diabetes Mellitus: Analysis of NHANES 2017–2018 and EPIC-Norfolk Cohorts

**DOI:** 10.3390/antiox12101863

**Published:** 2023-10-15

**Authors:** Anitra C. Carr, Helen Lunt, Nicholas J. Wareham, Phyo K. Myint

**Affiliations:** 1 Nutrition in Medicine Research Group, University of Otago, Christchurch 8011, New Zealand; 2 Diabetes Outpatients, Health New Zealand Waitaha Canterbury, Christchurch 8011, New Zealand; helen.lunt@cdhb.health.nz; 3 Department of Medicine, University of Otago, Christchurch 8011, New Zealand; 4 MRC Epidemiology Unit, Cambridge CB2 0QQ, UK; nick.wareham@mrc-epid.cam.ac.uk; 5 Ageing Clinical & Experimental Research (ACER) Team, Institute of Applied Health Sciences, University of Aberdeen, Aberdeen AB25 2ZD, UK; phyo.myint@abdn.ac.uk

**Keywords:** vitamin C, ascorbic acid, diabetes, diabetes mellitus, body weight, BMI, C-reactive protein, CRP, dietary intake, dietary requirements, NHANES, EPIC-Norfolk

## Abstract

Vitamin C is an essential enzyme cofactor and antioxidant with pleiotropic roles in human physiology. Circulating vitamin C concentrations are lower in people with diabetes mellitus, suggesting a higher dietary requirement for the vitamin. We interrogated the NHANES 2017–2018 and EPIC-Norfolk datasets to compare vitamin C requirements between those with and without diabetes mellitus using dose–concentration relationships fitted with sigmoidal (four-parameter logistic) curves. The NHANES cohort (n = 2828 non-supplementing adults) comprised 488 (17%) participants with diabetes (self-reported or HbA1c ≥ 6.5%). The participants with diabetes had a lower vitamin C status (median [IQR]) than those without (38 [17, 52] µmol/L vs. 44 [25, 61] µmol/L, *p* < 0.0001), despite comparable dietary intakes between the two groups (51 [26, 93] mg/d vs. 53 [24, 104] mg/d, *p* = 0.5). Dose–concentration relationships indicated that the group without diabetes reached adequate vitamin C concentrations (50 µmol/L) with an intake of 81 (72, 93) mg/d, whilst those with diabetes required an intake of 166 (126, NA) mg/d. In the EPIC-Norfolk cohort, comprising 20692 non-supplementing adults, 475 (2.3%) had self-reported diabetes at baseline. The EPIC cohort had a lower BMI than the NHANES cohort (26 [24, 28] kg/m^2^ vs. 29 [25, 34] kg/m^2^, *p* < 0.0001). Correspondingly, the EPIC participants without diabetes required a lower vitamin C intake of 64 (63, 65) mg/d while those with diabetes required 129 (104, NA) mg/d to reach adequate circulating vitamin C status. C-reactive protein concentrations were strongly correlated with body weight and BMI and provided a surrogate biomarker for vitamin C requirements. In conclusion, people with diabetes had 1.4 to 1.6 fold higher requirements for vitamin C than those without diabetes. This corresponds to additional daily vitamin C intake requirements of ~30–40 mg for people with diabetes, equating to a total daily intake of at least 125 mg/d.

## 1. Introduction

Normal human physiology has an absolute requirement for essential micronutrients (vitamins and trace elements) for adequate functioning of enzymes that rely on these micronutrients for their various activities. Vitamin C is one such essential micronutrient that is required through our diet, primarily from fresh fruits and vegetables, due to genetic mutations resulting in the loss of our ability to synthesise the nutrient in our livers [1]. Although best known for its antioxidant activities, being able to scavenge a wide range of reactive oxygen species, the vitamin has pleiotropic functions in human physiology through acting as a cofactor for a family of metalloenzymes that have numerous biosynthetic and regulatory functions [2,3,4]. As such, there has been significant interest in the role of vitamin C in both the prevention and treatment of various conditions, particularly those potentially modifiable by lifestyle and dietary changes, such as type 2 diabetes mellitus (T2DM) [5,6,7].

T2DM is a chronic health condition characterised by severe metabolic dysregulation and elevated morbidity and mortality [8,9]. Compared to healthy volunteers, vitamin C status is lower in people with T2DM, and the status of those with prediabetes and T1DM tends to be intermediate between that of healthy volunteers and those with T2DM [10,11]. There are several factors that could account for this observation in people with T2DM. Lower dietary intake of the vitamin may be anticipated, through avoidance of high sugar content fruit, for example. However, there is generally a trend towards improved dietary intake of the vitamin following a diagnosis of T2DM [12,13], and thus lower dietary intake does not appear to account for the lower vitamin C status observed [10,14]. Instead, likely factors include enhanced oxidative stress related to visceral fat-related inflammation [15], and a generally higher body weight resulting in a volumetric dilution of the vitamin [16]. Furthermore, enhanced renal leak of the vitamin is more prevalent in people with T2DM likely related to diabetic kidney dysfunction [17,18].

What is yet to be determined is how much extra vitamin C people with diabetes need to consume to compensate for the enhanced metabolic turnover, volumetric dilution and urinary loss of the vitamin. Recently, the analysis of vitamin C dose–concentration relationships has allowed for the estimation of vitamin C requirements based on various demographic and lifestyle factors, such as smoking and body weight [19]. Therefore, in the current study, we analysed data from two large international cohorts, the US National Health and Nutrition Examination Survey (NHANES) 2017–2018 and the European Prospective Investigation into Cancer (EPIC)-Norfolk study, to assess vitamin C dose–concentration relationships in people with diabetes to estimate how much extra vitamin C is required to reach an equivalent adequate circulating status (≥50 µmol/L) to those without diabetes. Due to the close associations of body weight and BMI with the inflammatory biomarker and cardiovascular disease risk factor C-reactive protein (CRP), the impact of CRP as a surrogate biomarker for vitamin C requirements was also explored.

## 2. Materials and Methods

### 2.1. NHANES 2017–2018 Cohort

**Figure 1:**
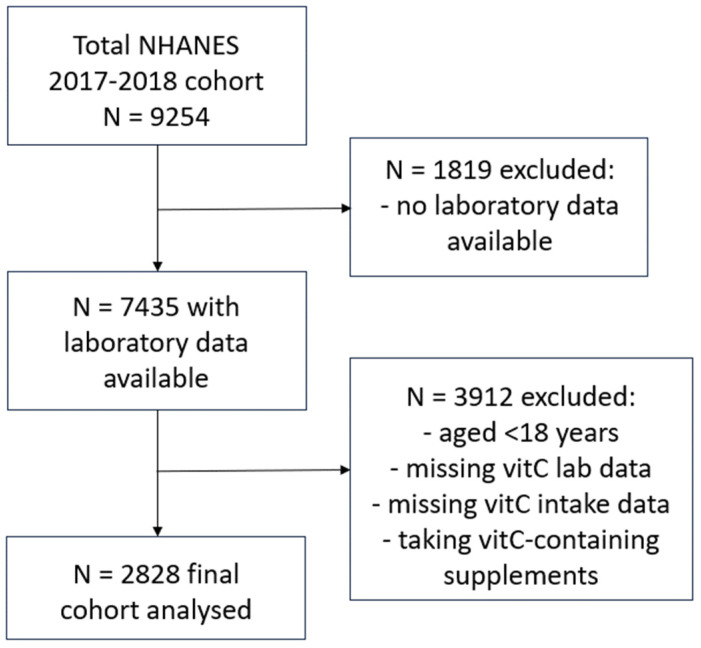
Participant selection flow diagram for NHANES 2017–2018 cohort.

NHANES uses a complex, multistage, probability sampling design to capture a representative sample of the US non-institutionalized civilian population. NHANES 2017–2018 data were extracted from the Centers for Disease Control and Prevention’s National Center for Health Statistics site: www.cdc.gov/nchs/nhanes/index.htm, as described previously [19]. Briefly, the final cohort (n = 2828) comprised adults aged 18 years or older, who had vitamin C laboratory values available, had completed two 24 h dietary recalls, and were not consuming vitamin C-containing supplements (Figure 1). Demographic and lifestyle variables collected for the current study included: age, sex, ethnicity, poverty income ratio, smoking status (including the number of years smoking and number of cigarettes smoked per day), body weight, BMI, waist-to-hip ratio and blood pressure. Dietary vitamin C intake (mg/d) was derived from the mean of two 24 h dietary recalls collected using the What We Eat In America Questionnaire and analysed using the USDA Food and Nutrient Database for Dietary Studies 2.0 (FNDDS 2.0). The first dietary recall interview was collected in person at the Mobile Examination Center and the second interview was collected by telephone 3 to 10 days later. Although vitamin C intake in the 24 h prior to blood collection most closely correlates with plasma vitamin C status, due to the vitamin’s water-soluble nature, the average of this and the second 24 h dietary recall was calculated to help normalize any non-typical (unusually high or low) daily intakes. Laboratory variables included: C-reactive protein concentrations, glycaemic indices (e.g., HbA1c, fasting blood glucose, insulin), lipid markers (e.g., triglycerides, LDL cholesterol, total cholesterol, HDL cholesterol), and the renal function parameters serum creatinine and urinary albumin to creatinine ratio (ACR). Non-fasting serum vitamin C concentrations (µmol/L) were measured using isocratic ultra-high performance liquid chromatography with electrochemical detection [20].

### 2.2. Assessment of Diabetes

The diabetes-related information comprised personal interview data on diabetes and the use of insulin or oral hypoglycemic medications. The diabetes-related questions were asked, in the home, by trained interviewers using a Computer-assisted Personal Interview (CAPI) system. Those with self-reported diabetes at baseline answered “yes” to the following question “Have you ever been told by a doctor or health professional that you have diabetes or sugar diabetes?” People with undiagnosed diabetes were identified using HbA1c concentrations ≥6.5%. The group with self-reported diabetes was further stratified into those with T2DM vs. T1DM using the treatment-based algorithm developed by Mosslemi et al. [21], which utilises the following questions: “Are you now taking insulin?”, “For how long have you been taking insulin?”, and “Are you now taking diabetic pills to lower your blood sugar?”, as well as the time since diagnosis to distinguish between T1DM and T2DM. People with prediabetes were identified using circulating HbA1c values between 5.7 and 6.4%. People with prediabetes were included in the non-diabetes group, unless otherwise stated.

### 2.3. EPIC-Norfolk Cohort

The EPIC-Norfolk study is a population-based prospective cohort study that recruited over 25,500 men and women aged 40–79 years at baseline between 1993 and 1997 from 35 participating general practices in Norfolk (https://www.epic-norfolk.org.uk (accessed on 23 April 2022). Individuals provided information about lifestyle behaviours, including diet and physical activity, and attended a baseline health check including the provision of blood samples and the collection of anthropometric data. The final cohort (n = 20,692) comprised participants who were not consuming vitamin C supplements and who had vitamin C laboratory values and complete 7-day food diary data available. Variables collected for the current study included: age, sex, smoking status (never, current, former), weight and BMI, vitamin C intake (derived from baseline 7-day food diaries [22,23]) and non-fasting plasma vitamin concentrations (analysed using a fluorometric assay [24]). People with established diabetes at baseline were defined as those who responded “yes” to the diabetes option of the question: “Has a doctor ever told you that you have any of the following?” followed by a list of conditions including diabetes, heart attack, and stroke.

### 2.4. Data Analyses

Median and interquartile range (Q1, Q3) were used for continuous variables, and counts with percentages were used for categorical variables. Group differences were assessed using non-parametric Mann–Whitney U tests with *p* values < 0.05 signifying statistical significance. Linear regressions were determined using Pearson coefficient and correlations using Spearman r. To estimate the vitamin C intakes required to reach ‘adequate’ serum vitamin C concentrations of 50 µmol/L and maximal steady-state serum concentrations attained at intakes of 200 mg/d, sigmoidal (four-parameter logistic) curves with asymmetrical 95% confidence intervals were fitted to vitamin C dose–concentration data. To calculate intake differences and relative requirements, the upper 95% CI of the first curve was related to the lower 95% CI of the second curve. Data were missing for smoking status for n = 79 (2.8%) participants in the NHANES cohort and n = 162 (0.8%) in the EPIC cohort; these participants were excluded from subgroup analyses stratified by smoking status. Data analyses and graphical presentations were carried out using GraphPad Prism 9 (GraphPad, San Diego, CA, USA).

## 3. Results

### 3.1. Characteristics of the NHANES Cohort Relative to Diabetes Status

The total NHANES cohort comprised 2828 non-supplementing adults with a median (IQR) age of 48 (32, 62) years. The cohort was further stratified relative to diabetes status: those who answered “yes” to the question “Have you ever been told by a doctor or health professional that you have diabetes or sugar diabetes?” (n = 393) or their HbA1c concentration was ≥6.5% if they had answered “no” to this question (n = 95), giving a total of 488 (17%) people with diabetes and 2340 (83%) without. Internationally, the prevalence of T2DM is much higher than that of T1DM and this difference is confirmed in the NHANES data. The group with self-reported diabetes (n = 393) was further stratified into those with T2DM vs. T1DM using the treatment-based algorithm developed by Mosslemi et al. [21]. Only a small number were identified as T1DM (n = 17; 0.6% of the total cohort), whereas 370 were identified as T2DM (with n = 24 of these being possible T2DM). The current study combined data from those with T1DM and T2DM into a single category of diabetes, unless otherwise stated. The median age of diabetes diagnosis was 50 (40, 58) years. The diabetes group was significantly older than the no-diabetes cohort (62 [52, 69] years vs. 43 [29, 60] years; *p* < 0.0001), and although fewer smoked in the diabetes group, those who smoked had been smoking for significantly longer (Table 1). The participants in the diabetes group were also significantly heavier and had a larger waist-to-hip ratio and a higher systolic blood pressure than those in the no-diabetes group (*p* < 0.0001).

Blood parameters also indicated significant differences between the diabetes and no diabetes groups (Table 2). Participants with diabetes had significantly higher C-reactive protein (CRP) concentrations, with the median value being in the high clinical risk category, i.e., >3 mg/L, as well as a higher percentage of HbA1c, and fasting blood glucose and fasting insulin concentrations (*p* < 0.0001). Significant differences were also observed in blood lipids (triglycerides and cholesterol) between the two groups (Table 2), as well as higher serum creatinine and urinary albumin to creatinine ratio (ACR), with >30% of the participants in the diabetes group exhibiting microalbuminuria (i.e., ACR > 30 mg/g).

### 3.2. Vitamin C Intake and Status Relative to Diabetes Status in the NHANES Cohort

**Figure 2:**
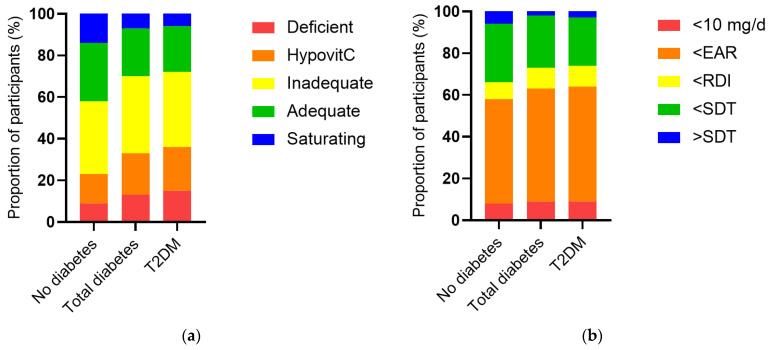
Serum vitamin C status and vitamin C intake categorised and stratified by diabetes status. (**a**) No diabetes n = 2340, total diabetes n = 488, T2DM n = 370. Serum vitamin C was categorised as deficient (≤11 µmol/L), hypovitaminosis C (≤23 µmol/L), inadequate (<50 µmol/L), adequate (≥50 µmol/L), and saturating (≥70 µmol/L). The total diabetes and T2DM groups had a significantly higher proportion of hypovitaminosis C and deficiency relative to the cohort without diabetes (*p* < 0.0001). (**b**) Vitamin C intake categorised as <10 mg/d, < estimated average requirement (EAR = 75 mg/d for males, 60 mg/d for females) [25], < recommended dietary intake (RDI = 90 mg/day for males, 75 mg/d for females) [25], < or > suggested dietary target (SDT = 220 mg/d for males and 190 mg/d for females) [26]. The total diabetes and T2DM groups had a slightly higher proportion of participants consuming less than the estimated average requirement relative to the cohort without diabetes (*p* = 0.04).

The vitamin C intake of the NHANES cohort was a median of 53 (24, 102) mg/d and this was associated with a less than adequate median vitamin C status of 43 (23, 60) µmol/L. When the cohort was stratified by diabetes status, the group with diabetes had significantly lower vitamin C status than the group without diabetes (38 [17, 52] µmol/L vs. 44 [25, 61] µmol/L, *p* < 0.0001), as well as a higher proportion of hypovitaminosis C and vitamin C deficiency (*p* < 0.0001, Figure 2a). This was despite a comparable dietary intake to the group without diabetes (51 [26, 93] mg/d vs. 53 [24, 104] mg/d, *p* = 0.5). The proportion of participants with an intake less than the estimated average requirement (EAR) was slightly higher in the group with diabetes (*p* = 0.04; Figure 2b). Comparable results were observed for the T2DM subgroup (Figure 2). Of note, there were significant inverse correlations between serum vitamin C and CRP (r = −0.190, *p* < 0.0001) and various glycaemic and lipid markers (Table 3), as well as the urine function markers serum creatinine (r = −0.188, *p* < 0.0001) and ACR (r = −0.077, *p* < 0.0001).

### 3.3. Vitamin C Requirements Stratified by Diabetes in the NHANES Cohort

**Figure 3:**
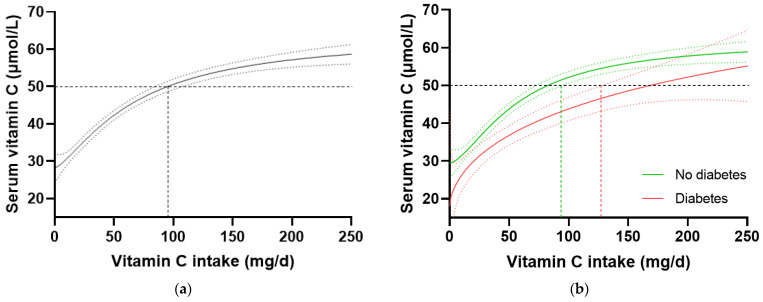
Serum vitamin C concentrations relative to vitamin C intake stratified by diabetes status in the NHANES cohort. (**a**) Total cohort n = 2828. (**b**) No diabetes group (green line) n = 2340; diabetes group (red line) n = 488. Sigmoidal (four-parameter logistic) curves were fitted to the dose–concentration data with asymmetrical 95% confidence intervals indicated. Dashed line indicates 50 µmol/L serum vitamin C which is considered ‘adequate’ [27].

In the total NHANES cohort (n = 2828), the intake of vitamin C required to reach ‘adequate’ serum concentrations (i.e., 50 µmol/L) was 97 (85, 106) mg/d (Figure 3a). When the cohort was stratified by diabetes status, the group without diabetes (n = 2340) reached 50 µmol/L serum vitamin C at an intake of 81 (72, 93) mg/d, whilst the group with diabetes (n = 488) required an intake of 166 (126, NA) mg/d (Figure 3b). Comparable results were observed when the T2DM subgroup was compared with the no-diabetes cohort. No differences in vitamin C requirements were observed for the subgroup with prediabetes (80 [63, 100] mg/d, n = 707) relative to those without prediabetes (81 [69, 96] mg/d, n = 1629). Since smoking is known to negatively affect the vitamin C dose–concentration relationship, resulting in higher vitamin C requirements in smokers, the participants who were current smokers (n = 681) and those without smoking status information (n = 79) were excluded, and the dose–concentration curves of non-smokers (n = 2069) were compared (Table 4). Although the curves were shifted to the left (i.e., requiring lower intakes to reach 50 µmol/L serum vitamin C), the higher requirement for vitamin C to reach adequate serum concentrations was still evident in the non-smoking participants with diabetes (n = 378) relative to the non-smoking participants without diabetes (n = 1960; Table 4). Overall, the participants with diabetes had a 1.4 fold higher requirement for vitamin C than those without diabetes, corresponding to an additional intake required to reach adequate vitamin C status being ~30 mg/d (the difference between the 95% CIs of the two curves), equating to a total daily intake of at least 125 mg/d.

### 3.4. Vitamin C Requirements Stratified by Diabetes in the EPIC-Norfolk Cohort

**Figure 4:**
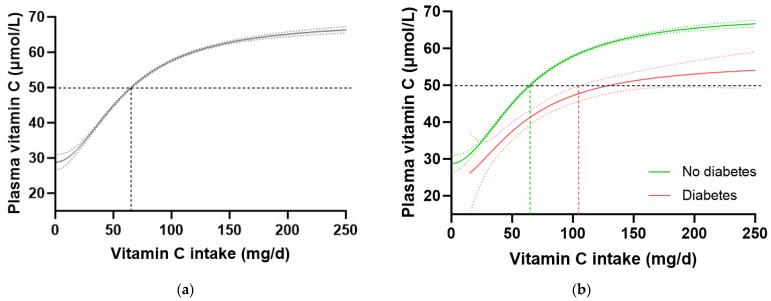
Plasma vitamin C concentrations relative to vitamin C intake stratified by diabetes status in the EPIC-Norfolk cohort. (**a**) Total cohort n = 20692. (**b**) No diabetes group (green line) n = 20193; diabetes group (red line) n = 475. Sigmoidal (four-parameter logistic) curves were fitted to the dose–concentration data with asymmetrical 95% confidence intervals indicated. Dashed line indicates 50 µmol/L serum vitamin C which is considered ‘adequate’ [27].

To confirm these findings in another cohort, we interrogated the EPIC-Norfolk dataset. This cohort was significantly larger (n = 20,692) than the NHANES cohort, but had a relatively lower proportion of participants with self-reported diabetes (n = 475, i.e., 2.3%). A number of other differences were apparent between the two cohorts. For example, the EPIC-Norfolk cohort was older (59 [51, 67] years), comprised a slightly higher proportion of females (53%), had a much lower prevalence of current smoking (11%), and a lower median weight (73 [64, 82] kg), BMI (26 [24, 28] kg/m^2^) and waist to hip ratio (0.86 [0.78, 0.93]), as well as a higher median vitamin C intake (76 [51, 112) mg/d) and correspondingly higher vitamin C status (53 [40, 64] µmol/L) than the NHANES cohort (Table 1; all *p* < 0.0001). Despite these differences, comparable trends in vitamin C requirements were observed in this cohort (Table 4). The total cohort required a vitamin C intake of 65 (64, 66) mg/d to reach adequate serum status (Figure 4a), and when stratified by diabetes status, the group without diabetes required an intake of 64 (63, 65) mg/d while the group with diabetes required 129 (104, NA) mg/d to reach equivalent status (Figure 4b). This corresponded to a 1.6 fold higher requirement for vitamin C for people with diabetes, equating to an additional intake requirement of ~39 mg/d to reach adequate serum concentrations of 50 µmol/L (Table 4). Furthermore, at a dietary intake of 200 mg/d, the people with diabetes did not reach as high a steady state concentration of vitamin C as those without (53 [49, 57] µmol/L vs. 65 [66, 64] µmol/L). Similar values were observed in the non-smoking cohort (n = 18,185) stratified by diabetes status (Table 4).

### 3.5. CRP as a Surrogate Biomarker for the Vitamin C Dose–Concentration Relationship

We have previously shown that body weight (and BMI) have a strong impact on both vitamin C concentrations and intake requirements in the NHANES cohort, with heavier BMI and weight tertiles having, respectively, 1.8 to 2 fold higher vitamin C requirements than the lighter BMI/weight tertiles [19]. Therefore, we assessed the associations between body weight and BMI with CRP to determine if CRP could be used as a surrogate biomarker for vitamin C intake requirements. There was a strong correlation between body weight and CRP concentrations in the total NHANES cohort (*r* = 0.408, *p* < 0.0001), corresponding to a 0.84 mg/L increase in CRP for every 10 kg in weight gain. Similarly, a strong correlation was found between BMI and CRP concentrations (*r* = 0.498, *p* < 0.0001), corresponding to a 0.78 mg/L increase in CRP for every 2.5 kg/m^2^ gain in BMI.

**Figure 5:**
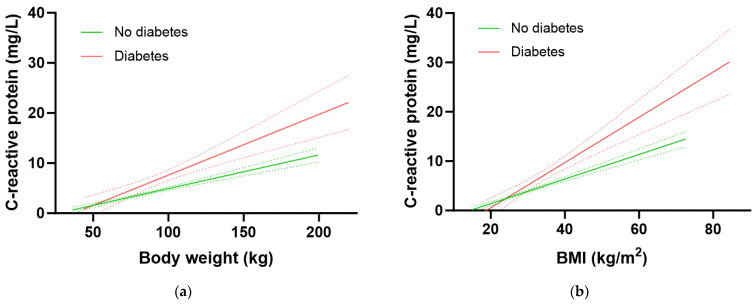
Association of C-reactive protein with body weight (**a**) and BMI (**b**). No diabetes group (green line) n = 2313; diabetes group (red line) n = 479; slopes were significantly different (*p* < 0.001). Dashed lines indicate 95% confidence intervals.

The participants with diabetes had significantly higher inflammation as assessed by CRP concentrations relative to participants without diabetes (3.2 [1.6, 7.1] mg/L vs. 1.8 [0.9, 4.3] mg/L, *p* < 0.0001). Furthermore, the group with diabetes had a significantly higher 1.21 mg/L increase in CRP for every 10 kg in weight gain relative to the group without diabetes (0.67 mg/L CRP per 10 kg weight gain; *p* = 0.0005; Figure 5a). Similarly, there was a 1.15 mg/L increase in CRP for every 2.5 kg/m^2^ gain in BMI in the group with diabetes relative to an increase of 0.63 mg/L CRP for every 2.5 kg/m^2^ gain in BMI in the group without diabetes (*p* < 0.0001; Figure 5b). Thus, the group with diabetes had higher CRP concentrations at equivalent BMIs (and weights) to those without diabetes. This elevated inflammation could be related to greater central obesity as evidenced by a higher waist-to-hip ratio in people with diabetes (0.99 [0.94, 1.04]) relative to those without (0.93 [0.87, 0.98]; *p* > 0.0001).

**Figure 6:**
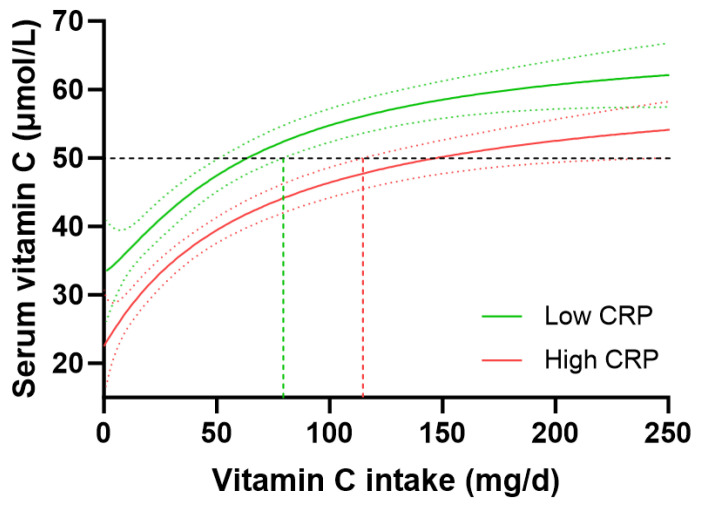
Impact of C-reactive protein (CRP) concentrations on the vitamin C dose–concentration relationship. Low CRP (<1 mg/L; green line) n = 765; high CRP (>3 mg/L; red line) n = 1081. The low CRP group had a median weight of 70 (61, 82) kg and BMI of 25 (22, 28) kg/m^2^; the high CRP group had a median weight of 89 (75, 108) kg and BMI of 33 (28, 38) kg/m^2^. Sigmoidal (four-parameter logistic) curves were fitted to the dose–concentration data with asymmetrical 95% confidence intervals indicated. Dashed line indicates 50 µmol/L serum vitamin C which is considered ‘adequate’ [27].

People with high-risk CRP concentrations (>3 mg/L, n = 1081) had significantly lower vitamin C concentrations than those with low-risk CRP concentrations (<1 mg/L, n = 765), i.e., 38 (20, 55) µmol/L vs. 50 (32, 65) µmol/L vitamin C (*p* < 0.0001). However, the higher-risk group also had significantly lower dietary vitamin C intakes (48 [21, 95] mg/d) than the lower-risk group (60 [27, 107] mg/d; *p* = 0.002). To take this into account, the vitamin C dose–concentration relationship for those with low CRP vs. high CRP was analysed. This indicated a vitamin C intake of 63 (51, 79) mg/d was required for people with low CRP to reach 50 µmol/L vitamin C concentrations relative to 146 (114, 229) mg/d for those with high CRP to reach equivalent plasma concentrations of the vitamin (Figure 6). People with medium CRP concentrations (1–3 mg/L) required vitamin C intakes of 91 (78, 113) mg/d to reach adequate circulating status. Overall, this equated to a 1.4 fold higher vitamin C requirement for people with high CRP vs. low CRP, corresponding to an increased vitamin C intake requirement of 35 mg/d in those with high CRP concentrations. Thus, stratifying the cohort by high vs. low CRP concentrations and computing the vitamin C dose–concentration relationship showed a comparable trend to the vitamin C requirements in people with diabetes relative to those without diabetes.

## 4. Discussion

In this study, we interrogated data from the NHANES 2017–2018 cohort and confirmed that people with diabetes had significantly lower vitamin C status than those without, despite consuming an equivalent dietary intake of the vitamin. This suggested that people with diabetes had a higher intake requirement for the vitamin. We then analysed vitamin C dose–concentration relationships using data from both the NHANES and EPIC-Norfolk cohorts to estimate how much additional vitamin C intake was required by those with diabetes. The data indicated a conservative 1.4 to 1.6 fold higher requirement for vitamin C in people with diabetes to reach comparable adequate vitamin C concentrations to those without diabetes. This corresponded to an additional daily vitamin C intake of ~30–40 mg in people with diabetes, equating to a total daily intake of at least 125 mg/d. Although smoking is well-known to affect the vitamin C dose–concentration relationship, resulting in higher intake requirements for smokers [19], this was accounted for in our study by also assessing non-smokers. Even though non-smokers had lower vitamin C requirements in general, the differences in vitamin C requirements between those with and without diabetes were comparable to the total cohorts, i.e., 1.4 to 1.7 fold higher vitamin C requirements for those with diabetes. Although the group with diabetes was older than the group without, we have previously shown that older age does not significantly impact the vitamin C intake required to provide adequate circulating concentrations [19]. Despite disparities in the baseline characteristics between the more recent (2017–2018) US NHANES cohort and the older (1993–1997) UK EPIC-Norfolk cohort, the differences in vitamin C requirements between the groups with and without diabetes were comparable, suggesting a common link between vitamin C requirements and diabetes status.

Body weight has a large impact on vitamin C requirements due to a larger volume requiring a higher dose to reach the same concentration as that observed for a smaller volume [16]. Vitamin C’s enzyme cofactor and antioxidant functions rely on suitably high concentrations for optimal activity [28]. The group with diabetes had a significantly higher body weight and BMI than those without diabetes and we have previously shown in the NHANES cohort that higher BMI and body weight tertiles resulted in 1.8 to 2 fold higher requirements for vitamin C than the lower BMI and body weight tertiles [19]. In the current study, we observed a strong correlation between CRP concentrations and both body weight and BMI, which was even more apparent in the group with diabetes at equivalent weights and BMI. Analysis of the vitamin C dose–concentration relationship stratified by CRP concentrations of low (<1 mg/L) vs. high (>3 mg/L) risk for cardiovascular disease indicated a 1.4 fold higher vitamin C requirement for those with high CRP, corresponding to an increased daily vitamin C intake requirement of 35 mg, for a total daily intake of at least 115 mg/d. As such, people with high CRP (>3 mg/L) had comparable vitamin C requirements to people with diabetes; thus, CRP appears to be a suitable surrogate biomarker for determining vitamin C requirements. Since elevated inflammation has been implicated in attenuated intestinal vitamin C uptake [29,30], this could contribute to a requirement for higher vitamin C intake in people exhibiting elevated systemic inflammation.

Of consideration, inverse associations previously reported between vitamin C and high-sensitivity CRP [31], could have BMI or body weight as the common factor, with CRP acting as a proxy biomarker for higher BMI or body weight. Thus, studies showing an impact of vitamin C on high-sensitivity CRP may be confounded by changes in the participant’s body weight. As such, the relationships between vitamin C, high-sensitivity CRP and body weight or BMI need to be investigated in more detail in future studies.

People with greater abdominal fat mass tend to exhibit elevated inflammatory biomarkers and associated oxidative stress [15]. In the NHANES and EPIC-Norfolk cohorts, the people with diabetes had significantly higher waist-to-hip ratios indicating greater abdominal obesity in this group. Thus, people with diabetes and associated higher abdominal fat mass may be expected to have a greater turnover of vitamin C through its antioxidant activities [32]. Research has also indicated a greater loss of vitamin C in people with diabetes via enhanced renal leak, likely associated with diabetic kidney dysfunction [17,18]. We observed a weak inverse correlation between vitamin C concentrations and ACR in the NHANES cohort suggesting possible loss of vitamin C with increasing renal dysfunction. Thus, the enhanced requirements for vitamin C in the group with diabetes likely comprises a combination of higher body weight and enhanced renal dysfunction, with a likely contribution by elevated oxidative stress. Since higher dietary intakes and circulating concentrations of vitamin C have been associated with a lower risk of diabetes morbidity and mortality [6,7], increasing vitamin C intake in people with diabetes to account for their higher requirements may help attenuate the progression of the disease to more severe complications. In support of this premise, meta-analyses of intervention studies have indicated that supplementation with vitamin C can improve dysregulated glycaemic and lipid markers and cardiovascular risk factors in people with diabetes [33,34,35]. Furthermore, preliminary research has indicated the potential benefit of vitamin C in diabetic foot disease [36,37].

Our study findings have important implications for the setting of dietary intake recommendations for vitamin C. The current recommended dietary allowance (RDA) for vitamin C in the US is 90 mg/d for men and 75 mg/d for women [25]. The RDA is meant to meet the vitamin C intake needs of 97.5% of the adult population, with the caveat that there are specific subgroups within the population who have higher intake needs, for example, pregnant and lactating women and smokers [28]. Although women have a lower RDA based on their lower body weight, there is currently no specific intake category for people with higher body weight [28], despite the looming obesity pandemic [38]. In the NHANES cohort, nearly one in five participants had diabetes, and since body weight/obesity is a factor in the higher vitamin C requirements of people with diabetes, this suggests that a large proportion of the US population is not being adequately provided for by the current US RDAs for vitamin C (which were published more than 20 years ago) [25]. As such, the introduction of a new vitamin C intake category based on higher body weight or higher BMI would better provide for not only those who are overweight/obese, but also those with diabetes.

A limitation of the current research is the non-fasting vitamin C samples. Fasting samples would improve the accuracy of the vitamin C requirement estimates; however, a requirement for fasting samples can be challenging for the recruitment of participants in large epidemiological studies. Another limitation is the relatively small size of the diabetes groups in the two cohorts, resulting in relatively large 95% CIs and a more conservative estimate of vitamin C requirements. Thus, larger cohorts of people with diabetes would decrease the 95% CIs and thus increase the difference in requirements between those with and without diabetes, as was observed to a certain extent with the larger EPIC-Norfolk cohort relative to the NHANES cohort. As such, the true vitamin C requirements for people with diabetes may be significantly greater than the 1.6 fold currently estimated.

## 5. Conclusions

Our research has shown a clear link between diabetes and a higher requirement for vitamin C, with a conservative estimate of 1.4 to 1.6 fold higher vitamin C intake requirement for people with diabetes to reach adequate circulating concentrations of the vitamin. This corresponds to an additional daily vitamin C intake of ~30–40 mg for people with diabetes, equating to an total daily intake of at least 125 mg/d. These findings have important implications for the setting of global vitamin C dietary intake guidelines. Future dose-finding studies are needed to definitively establish how much vitamin C is required by people with diabetes to compensate for their higher requirements for the vitamin.

## Figures and Tables

**Table 1:** NHANES cohort characteristics relative to diabetes status.

Characteristics	Total Cohort (n = 2828)	No Diabetes (n = 2340)	Diabetes (n = 488)	*p* Value ^1^
Age, years	48 (32, 62)	43 (29, 60)	62 (52, 69)	<0.0001
Sex, n (%)				
Male	1425 (50)	1171 (50)	254 (52)	
Female	1403 (50)	1169 (50)	234 (48)	0.4
Ethnicity				
Non-Hispanic White	941 (33)	801 (34)	140 (29)	
Non-Hispanic Black	727 (26)	582 (25)	145 (30)	
Mexican American	399 (14)	316 (13)	83 (17)	
Non-Hispanic Asian	328 (12)	283 (12)	45 (9)	
Other Hispanic	281 (10)	235 (10)	46 (9)	
Other/multi-race	152 (5)	123 (5)	29 (6)	0.7
Poverty income ratio	1.9 (1.1, 3.8)	2.0 (1.1, 3.8)	1.9 (1.1, 3.7)	0.7
Current smoker ^2^	681 (25)	588 (26)	93 (20)	0.006
No. of years smoking	34 (19, 47)	30 (17, 46)	44 (35, 52)	<0.0001
No. of cigarettes/day	8 (4, 15)	8 (4, 15)	10 (4, 20)	0.2
Body weight, kg	80 (68, 97)	79 (67, 95)	91 (75, 105)	<0.0001
Body mass index, kg/m^2^	29 (25, 34)	28 (24, 33)	32 (28, 37)	<0.0001
Waist/hip ratio	0.94 (0.88, 0.99)	0.93 (0.87, 0.98)	0.99 (0.94, 1.04)	<0.0001
Systolic BP, mmHg	123 (111, 135)	121 (111, 133)	130 (119, 145)	<0.0001
Diastolic BP, mm Hg	73 (65, 80)	73 (65, 81)	72 (65, 79)	0.5
Vitamin C intake, mg/d	53 (24, 102)	53 (24, 104)	51 (26, 93)	0.5

Data represent median (Q1, Q3) or n (%). ^1^ *p* value is for diabetes vs. no diabetes. ^2^ Data were missing for smoking status for 79 (2.8%) participants.

**Table 2:** Biochemical parameters of the NHANES cohort.

Characteristics	Total Cohort (n = 2828)	No Diabetes (n = 2340)	Diabetes (n = 488)	*p* Value ^1^
C-reactive protein, mg/L	2.1 (0.9, 4.8)	1.8 (0.9, 4.3)	3.2 (1.6, 7.1)	<0.0001
HbA1c, %	5.5 (5.2, 5.9)	5.4 (5.2, 5.7)	7.0 (6.4, 8.2)	<0.0001
FBG, mmol/L ^2^	5.7 (5.3, 6.3)	5.6 (5.3, 6.0)	8.3 (6.5, 10)	<0.0001
Insulin, pmol/L ^3^	61 (38, 99)	57 (37, 92)	86 (52, 145)	<0.0001
Triglycerides, mmol/L	1.0 (0.7, 1.5)	1.0 (0.7, 1.4)	1.4 (1.0, 1.9)	<0.0001
LDL cholesterol, mmol/L	2.8 (2.3, 3.5)	2.9 (2.3, 3.5)	2.6 (1.9, 3.2)	<0.0001
Total cholesterol, mmo/L	4.7 (4.1, 5.5)	4.7 (4.1, 5.5)	4.7 (3.9, 5.4)	0.004
HDL cholesterol, mmol/L	1.3 (1.1, 1.6)	1.3 (1.1, 1.6)	1.1 (1.0, 1.4)	<0.0001
Serum creatinine, µmol/L	74 (63, 88)	74 (62, 88)	78 (62, 95)	0.005
Urinary ACR, mg/g	7.3 (4.7, 13.9)	6.7 (4.6, 12)	13 (6.9, 44)	<0.0001
Vitamin C, µmol/L	43 (23, 60)	44 (25, 61)	38 (17, 52)	<0.0001

Data represent median (Q1, Q3) or n (%). ^1^ *p* value is for diabetes vs. no diabetes. ^2^ FBG, fasting blood glucose, n = 1355. ^3^ Fasting insulin, n = 1337 (of these, 63 were taking insulin: 4.7% of the total group and 26% of the diabetes subgroup). ACR, albumin to creatinine ratio.

**Table 3:** Correlations between serum vitamin C and blood biomarkers in the NHANES cohort.

Characteristics	Spearman *r*	*p* Value
C-reactive protein, mg/L	−0.190	<0.0001
HbA1c, %	−0.093	<0.0001
FBG ^1^, mmol/L	−0.106	<0.0001
Insulin, pmol/L	−0.114	<0.0001
Triglycerides, mmol/L	−0.102	0.0002
LDL cholesterol, mmol/L	−0.044	0.11
Total cholesterol, mmo/L	−0.029	0.12
HDL cholesterol, mmol/L	0.168	<0.0001
Serum creatinine, µmol/L	−0.188	<0.0001
Urinary ACR ^2^, mg/g	−0.077	<0.0001

^1^ FBG, fasting blood glucose; ^2^ ACR, albumin to creatinine ratio.

**Table 4:** Vitamin C intakes required to reach ‘adequate’ serum concentrations.

Datasets (n)	Curve 1 Intake (mg/d) ^1^	Curve 2 Intake (mg/d) ^1^	Δ Intake (mg/d)	Δ 95% CI (mg/d)
NHANES total (n = 2828)	97 (85, 106)			
No diabetes (n = 2340)	81 (72, 93)			
Diabetes (n = 488)		166 (126, NA)	85	33
Non-smokers (n = 2068)	76 (67, 85)			
No diabetes (n = 1960)	64 (57, 74)			
Diabetes (n = 378)		145 (102, NA)	81	28
EPIC total (20,692)	65 (64, 66)			
No diabetes (n = 20,193)	64 (63, 65)			
Diabetes (n = 475)		129 (104, NA)	65	39
Non-smokers (n = 18,185)	62 (61, 63)			
No diabetes (n = 17,734)	61 (60, 62)			
Diabetes (n = 439)		126 (104, 164)	65	42

^1^ Estimated vitamin C intakes required to reach 50 µmol/L serum vitamin C concentrations. Data was estimated from sigmoidal (four-parameter logistic) curves with asymmetrical 95% confidence intervals fitted to dose–concentration data. NA, not attainable (i.e., did not reach 50 µmol/L).

## Data Availability

The NHANES data used in the manuscript is publicly and freely available without restriction at https://www.cdc.gov/nchs/nhanes/index.htm (accessed on 7 December 2022). The EPIC data is available by request through https://www.epic-norfolk.org.uk/for-researchers/data-sharing/data-requests (accessed on 4 April 2022).
